# Health Officer at the Port of N. York

**Published:** 1848-03

**Authors:** 


*Health Officer at the Port of N. York.*—This lucrative office, reputed to be worth over $20,000 *per annum*, has been conferred by the Governor and Senate, upon Dr. Whiting, of the city of New-York. The Annalist states, that, pending the confirmation of the nomination of Dr. Whiting by the Governor, Dr. Van Hoevenburg, the former incumbent, resigned, and Dr. Samuel R. Childs was appointed by the Board of Health to fill the vacancy. The latter immediately entered upon the duties, and still continues to perform them. An important legal question is involved in these proceedings, about which there are various opinions, the revised Constitution not providing a solution of the difficulty. Our contemporary adds, that Dr. C. has already made himself very popular in his new office, and it is presumed that the continuance of the present arrangement would give entire satisfaction.

